# The TGFβ-Induced Long Non-coding RNA TBULC Promotes the Invasion and Migration of Non-small Cell Lung Cancer Cells and Indicates Poor Prognosis

**DOI:** 10.3389/fonc.2019.01340

**Published:** 2019-12-10

**Authors:** Sufei Zheng, Zhiliang Lu, Chengming Liu, Xinfeng Wang, Runsen Jin, Shuangshuang Mao, Jianbing Huang, Yuanyuan Lei, Chaoqi Zhang, Nan Sun, Jie He

**Affiliations:** Department of Thoracic Surgery, National Cancer Center/National Clinical Research Center for Cancer/Cancer Hospital, Chinese Academy of Medical Sciences and Peking Union Medical College, Beijing, China

**Keywords:** non-small cell lung cancer, TGFβ, long non-coding RNA, TBULC, prognosis

## Abstract

**Objective:** To investigate the biological function and clinicopathological significance of the TGFβ-induced long non-coding RNA (lncRNA) TBULC in non-small cell lung cancer (NSCLC) and to analyze its potential value in clinical diagnosis and treatment.

**Methods:** RT-qPCR was used to detect the expression level of TBULC in NSCLC cells and tissues, and the correlation between the TBULC expression level and clinicopathological characteristics was analyzed. A cytoplasmic/nuclear fractionation assay was performed to define the cellular localization of the TBULC. A rapid amplification of cDNA ends (RACE) assay was performed to acquire the full-length sequence of the TBULC. Stable TBULC overexpression and TBULC knockdown cell clones were constructed by lentiviral infection, and Transwell assays were used to explore the effect of the TBULC on cell invasion and migration.

**Results:** Stimulation with TGFβ in NSCLC cell lines significantly upregulated the expression level of the nuclear-localized lncRNA TBULC. The RACE assay indicated that the full-length TBULC sequence was 1,020 nucleotides, and the sequence was located on chromosome 15. Cell function experiments showed that the TBULC played a crucial role in promoting NSCLC metastasis. Knockdown of TBULC significantly suppressed the invasion and migration of NSCLC cells, and overexpression of TBULC had the opposite effects. The expression level of TBULC in 106 NSCLC tumor tissues was significantly higher than that in adjacent normal tissues, and TBULC was proven to be an independent prognostic factor in NSCLC patients [*p* = 0.030, OR = 0.513 (0.281–0.936)].

**Conclusion:** The TGFβ-induced lncRNA TBULC was upregulated in NSCLC and promoted the invasion and migration of NSCLC cells. TBULC was an independent prognostic factor and might be a potential biomarker for predicting the prognosis of NSCLC patients.

## Introduction

Lung cancer is the leading cause of cancer death worldwide, with ~2.1 million new cases and 1.8 million mortalities worldwide each year (1). Non-small cell lung cancer (NSCLC) accounts for 85% of lung cancers, with lung adenocarcinoma (LUAD) and lung squamous cell carcinoma (LUSC) as the predominant pathologic subtypes (2). Despite improvements in the diagnosis and treatment of NSCLC in recent decades, the 5-year survival rate of NSCLC remains dismal at 15% owing to the recurrence caused by local diffusion and metastasis (3). Thus, further investigations into the mechanisms of metastasis and relapse and the development of novel treatment strategies for NSCLC are still required to improve NSCLC clinical outcomes.

Previous studies have shown that the transforming growth factor beta (TGFβ) pathway regulates epithelial-mesenchymal transition (EMT), angiogenesis, and immune escape and plays crucial roles in cancer by promoting proliferation, invasion, and metastasis (4–6). Owing to the vital role of this pathway in cancer, many drugs targeting genes encoding components of the TGFβ pathway have been developed in recent decades (5, 7, 8); however, the application of these targeted drugs in clinic has been hindered by the dual roles of the TGFβ signaling pathway in the suppression and promotion of cancer (4, 9, 10). Although the genes encoding components of the TGFβ signaling pathway have been well-studied in recent decades, the contextual complexity of the TGFβ signaling pathway remains unclear.

Recent studies suggest that long non-coding RNAs (lncRNAs), which are RNAs >200 nucleotides in length that have no coding potential and represent the majority of non-coding RNAs (11, 12), are extensively involved in the initiation and progression of cancer. Moreover, they are often highly dysregulated in malignant tumors (11, 13–15). The latest evidence has shown that several lncRNAs involved in the TGFβ signaling pathway extensively participated in mediating EMT and NF-κB pathway activation and promoted the invasion-metastasis cascade in various cancers (16–18). Additionally, our previous study demonstrated that the TGFβ-induced lncRNA TBILA was highly expressed in NSCLC and promoted cell invasion and migration (6). We also found a large number of previously unexplored TGFβ-induced lncRNAs (19), among which TGFβ Upregulated lncRNA in Lung Cancer (TBULC), one of the most prominent lncRNAs, was markedly upregulated in lung cancer compared with normal lung tissues. Nevertheless, its biological functions and underlying molecular mechanisms in lung cancer remain largely unclear and require elucidation.

In this study, we aimed to investigate the contributions of the lncRNA TBULC to the TGFβ signaling pathway in NSCLC and focused on its role in the invasion-metastasis cascade of NSCLC. In addition, the present study has provided a more detailed understanding of TBULC as a novel potential therapeutic target in NSCLC patients.

## Materials and Methods

### Patient Tissue Samples and Cell Lines

A total of 106 paired NSCLC tumors and adjacent normal tissues were used. All patients underwent radical surgical therapy in the Department of Thoracic Surgery of the Cancer Hospital of the Chinese Academy of Medical Sciences between November 2011 and December 2012. None of the patients received radiotherapy or chemotherapy before surgery. There were no other tumors or antitumor treatments within 3 years of the surgery. The TNM stage was classified according to the eighth edition of the American Joint Committee on Cancer (AJCC) lung cancer staging system. The tumor tissues and adjacent normal tissues of these 106 patients were snap frozen in liquid nitrogen immediately after resection and stored at −80°C until they were used in this study. All specimens were confirmed by pathology as LUAD or LUSC, and all patients provided informed consent before surgery.

NSCLC cell lines (A549 and H226) were obtained from the American Type Culture Collection (ATCC). The cancer cell lines were maintained in RPMI 1640 supplemented with 10% fetal bovine serum (FBS) (Gibco) and 1% antibiotics (100 U/ml penicillin and 100 mg/ml streptomycin) (Invitrogen). All cell lines were cultured at 37°C in a 5% CO_2_ cell culture incubator (Thermo). The cells were grown for no more than 25 passages for any experiment. The identities of all cell lines were confirmed by matching the short tandem repeat (STR) profile to the registered information in the Department of Human and Animal Cell Cultures (DSMZ) online STR database. TGF-β1 was purchased from R&D and used at a 5 ng/ml concentration. The treatment time was 24 h unless specified. To inhibit TGF-β signaling, 5 μM SB505124 (Selleck) was added to the culture medium 30 min prior to the specified treatments.

### RNA Extraction, RT-qPCR, and Transwell Assays

RNA extraction, RT-qPCR, and Transwell assays were performed as described previously (20). In brief, total RNA was extracted with TRIzol Reagent (Sigma), and cytoplasmic and nuclear RNA was isolated and purified using the Protein and RNA Isolation System (Life Technologies). A RevertAid First-Strand cDNA Synthesis kit (Thermo Scientific) was used for cDNA synthesis, and qPCR was performed on the cDNA using a SYBR Green Mix (Thermo Scientific) and a real-time PCR machine. The PCR primer sequences are listed: GAPDH (sense: CCT GGT ATG ACA ACG AAT TTG; antisense: CAG TGA GGG TCT CTC TCT TCC) and TBULC (sense: GGG GTG AGA GGA ACA ACA AA; antisense: TGT CAG GGG AGC CAT ACA C). Transwell assays were performed to detect the migration and invasion ability of cells by using Transwell chambers coated with or without Matrigel (BD Biosciences).

### Chromatin Immunoprecipitation (ChIP)

The Smad 2/3 binding motifs were predicted by JASPAR (http://jaspar.genereg.net/). ChIP was performed using the Simple ChIP Enzymatic Chromatin Immunoprecipitation Kit (#9003, CST) strictly according to the instructions. Briefly, cross-linked chromatin was enzymatically digested into 150- to 900-bp fragments (1–5 nucleosomes). The chromatin fragments were immunoprecipitated by an anti-Smad2/3 antibody. Normal mouse immunoglobulin G (IgG) was used as a negative control. The chromatin immunoprecipitated by the Smad2/3 antibody was then analyzed by RT-qPCR using specific primers for the promoter region of TBULC (sense: AGG CTT GGC AGT TTC CTT ACC C; antisense: AGG CTT GGC AGT TTC CTT ACC C).

### Rapid Amplification of cDNA Ends (RACE)

To determine the transcription of the TBULC start and stop sites, 5′-RACE and 3′-RACE were performed using the SMARTer™ RACE cDNA Amplification Kit (Clontech, Palo Alto, CA). The 5′-RACE and 3′-RACE products of nested PCR were linked by the pEASY-Blunt Zero Cloning Kit (Transgen, Beijing, China) according to the manufacturer's instructions. This was followed by Sanger sequencing to obtain the 5′ and 3′ sequences. The nested PCR primer sequences were as follows: 3'-RACE primer1: CCC CAA ACG CTG GGG TGA GAG GAA C; 3′-RACE primer2: CTG TCA AAC ACA TGG TGT ATG GCT CCC C; 3'-RACE primer3: GAG CAT ACA CTG TGG GAA GGC TCA GCT G; 5′-RACE primer1: CCA GTC TCA GAC TTC CAA GAA TGC GCG; 5′-RACE primer2: CAG CTG AGC CTT CCC ACA GTG TAT GCT C; and 5′-RACE primer3: GCC GGC ACA AAG TTG CAG TTA TAC AGG A.

### Construction of Cell Lines With Stable Overexpression or Knockdown of TBULC

The full-length gene sequence (1020 bp) of TBULC was synthesized and cloned into the pCDH-CMV-MCS-EF1-GFP+Puro (CD513B-1) vector (Generay, Shanghai, China). The shRNA sequence was ligated into the pLKO.1 plasmid for viral packaging and infection to construct TBULC-silenced cell clones. The shRNA sequences were shRNA1, GCA GAT GGT AAA CAA ACA TGC and shRNA2, GCT GTC CTC TTA TGA CAA TAT. Lentiviral packaging and infection were performed as described previously (6). Virus particles in cells were harvested 48 h after CD513B-1-TBULC transfection using Lipofectamine 3000 reagent (Life Technologies). A549 and H226 cells were infected with the recombinant lentivirus-transducing units with 8 mg/ml polybrene (Sigma-Aldrich) to increase the efficiency of infection.

### Statistical Analysis

All statistical analyses were performed using SPSS Ver 24.0 software (IBM, USA) or GraphPad Prism 8 (GraphPad Software, Inc.). Measurement data were compared using an independent sample Student's *t*-test. A paired Student's *t*-test was used to compare the expression level of TBULC between the tumor and adjacent normal tissues. The correlation between TBULC and clinicopathological features was analyzed by the chi-square test. Survival curves were plotted by the Kaplan–Meier method and compared by the log-rank test. The significance of various variables for survival was analyzed by the Cox proportional hazards model for multivariate analyses. A probability value of 0.05 or less was considered to be statistically significant.

## Results

The lncRNA TBULC was upregulated by the classical TGFβ/Smad signaling pathway.

**Figure 1:**
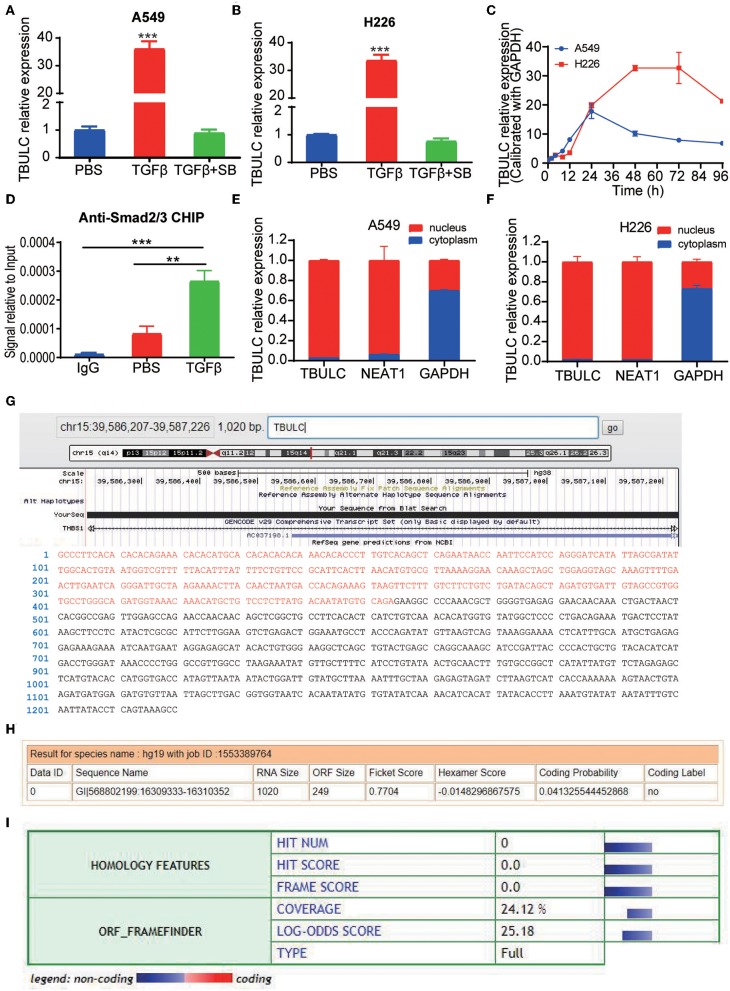
TBULC was upregulated by the classic TGF-β pathway. **(A,B)** Relative expression levels of TBULC in cancer cell lines **(A)** A549and **(B)** H226 treated with phosphate-buffered saline (PBS), TGF-β1, or TGF-β1 plus the TGF-β inhibitor SB505124 (SB), as measured by RT-qPCR. **(C)** Relative expression levels of TBULC in A549 and H226 cells at 4, 8, 12, 24, 48, 72, and 96 h after TGF-β1 stimulation. **(D)** The Smad2/3 complex localized to the TBULC promoter in A549 cells treated with TGF-β1 or PBS for 30 min, as determined by the ChIP assay. **(E,F)** Subcellular localization of TBULC in **(E)** A549 and **(F)** H226 cells, as assessed by RT-qPCR; GAPDH and NEAT1 RNA were used as fractionation indicators. **(G)** Graphic depiction of the full-length sequence of TBULC, which was obtained by RACE. **(H,I)** The online coding potential analysis tool **(H)** Coding Potential Calculator (CPC) and **(I)** Coding Potential Assessment Tool (CPAT) confirmed that TBULC was a non-coding RNA with no coding potential. The data are shown as the mean ± SD, **p < 0.01, ***p < 0.001.

In previous studies, we compared lncRNA expression in the A549 and H226 cell lines with or without TGFβ1 treatment continuously for 72 hours by transcriptome sequencing. CTD-2033D15.2 (ENST00000478845.2), one of the lncRNAs with greatest increase in expression, was upregulated by more than 20 times in both TGFβ-treated cell lines (6). As CTD-2033D15.2 was upregulated in TGFβ-treated lung cancer cells, we named it TGFβ Upregulated lncRNA in Lung Cancer (TBULC). The induction of TBULC was verified by RT-qPCR in repeated independent experiments, and the upregulation of TBULC induced by TGFβ1 treatment could be completely abolished by the TGFβ pathway-specific small molecule inhibitor SB505124 (Figures 1A,B). Metabolic kinetic assays revealed that the expression level of TBULC peaked at 24 and 48 h after TGFβ treatment in A549 and H226 cells, respectively, and was maintained at high levels for at least 96 h (Figure 1C). To determine whether TBULC is directly modulated by the TGFβ/Smad classical pathway, we investigated the promoter region of TBULC via the online tool JASPAR and found several Smad2/3 transcription factor complex binding sites. According to these binding sites, we designed specific PCR primers to detect the anti-Smad2/3 antibody-immunoprecipitated chromatin fragments. The results showed that the TBULC promoter region was significantly enriched by the anti-Smad2/3 antibody rather than the IgG control. Interestingly, this enrichment was more obvious after TGFβ1 stimulation, indicating that TBULC was directly regulated by TGFβ/Smad classical signaling (Figure 1D). In addition, the mechanism by which lncRNA exerts its function is greatly affected by its cell localization. Thus, we compared the expression levels of TBULC in the nucleus and cytoplasm using RT-qPCR and clarified that TBULC was mainly localized in the nucleus (Figures 1E,F). Furthermore, the lncRNA TBULC has not been previously studied, and its transcription initiation and termination sites are unknown. We designed primers for the RACE assay and obtained a full-length sequence of 1,020 nucleotides, which was mapped on chromosome 15, and we highlighted additional sequences identified by RACE that are different from the TBULC sequence submitted to the public database (Figure 1G). To determine whether TBULC has coding potential, we used the online coding potential analysis tool Coding Potential Calculator (CPC, http://cpc.cbi.pku.edu.cn/) as well as the Coding Potential Assessment Tool (CPAT, http://lilab.research.bcm.edu/cpat/) to analyze the full-length sequence of TBULC and confirmed that TBULC was a non-coding RNA with no coding potential (Figures 1H,I).

### TBULC Promotes the Invasion and Migration of NSCLC Cells *in vitro*

**Figure 2:**
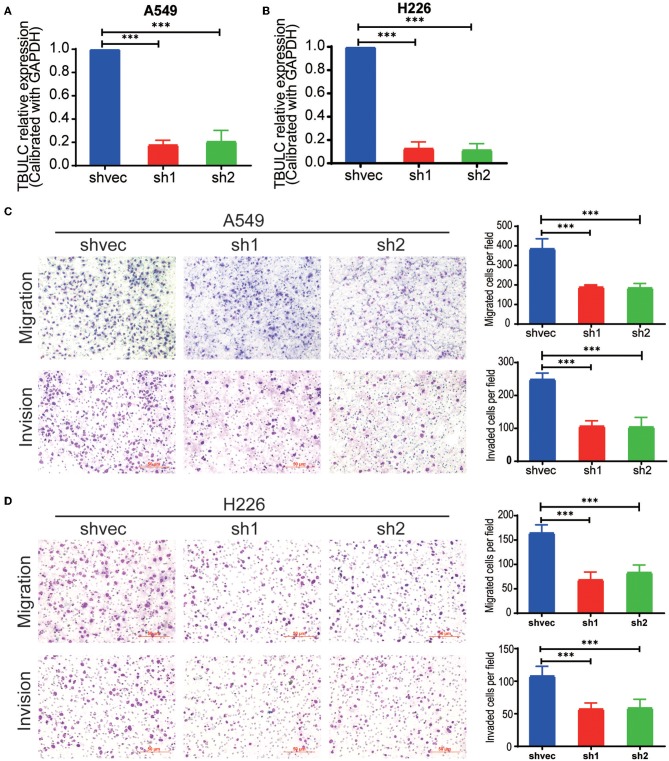
TBULC knockdown inhibited cell migration and invasion *in vitro*. **(A,B)** The expression level of TBULC in stable TBULC knockdown or mock vehicle control-transfected **(A)** A549 and **(B)** H226 cell clones. **(C,D)** The migration and invasion ability of TBULC knockdown **(C)** A549 and **(D)** H226 cell clones, as detected by the Transwell assay. The numbers of migrating and invading cells were compared between the groups. The data are shown as the mean ± SD. ***p < 0.001.

To investigate the role of TBULC in NSCLC cells, we silenced the expression of TBULC in A549 and H226 NSCLC cells with shRNA. As shown in Figures 2A,B, in both knockdown clones, the expression level of TBULC was markedly downregulated by more than 70%. A Transwell assay was employed to determine whether cell invasion and migration were affected by TBULC in NSCLC cells. The results showed that the invasive activity of silenced cell clones passing through Matrigel-coated membranes was significantly inhibited compared with that of the control cells (Figures 2C,D). Moreover, consistent with the invasion assay results, TBULC silencing also significantly suppressed the migration capacity of NSCLC cells (Figures 2C,D).

**Figure 3:**
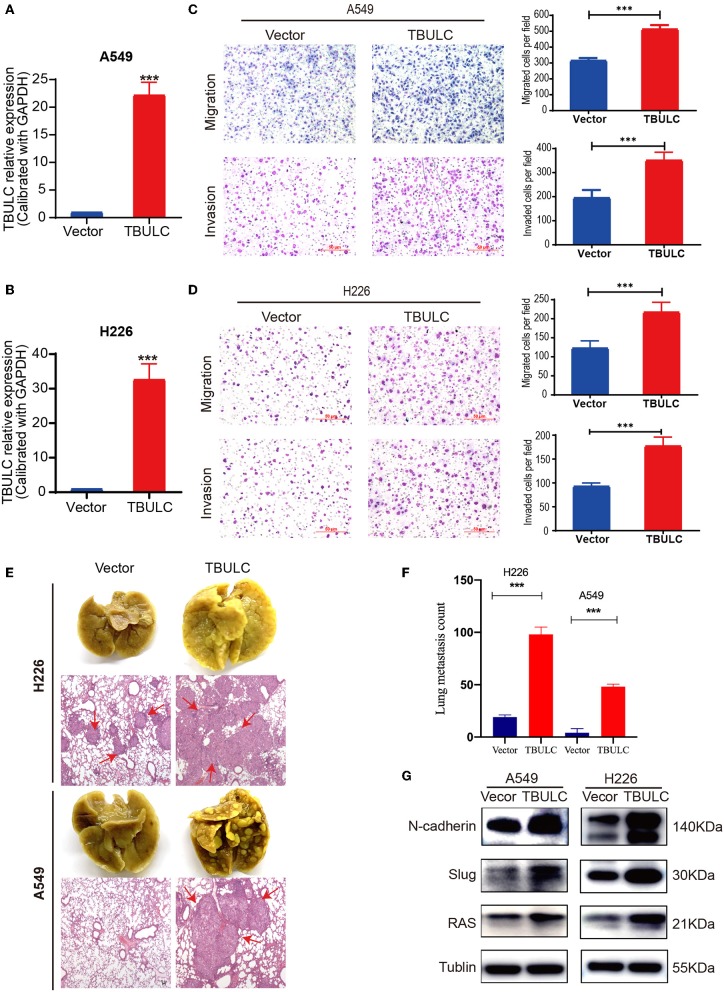
TBULC overexpression promoted cell migration and invasion *in vitro* and *in vivo*. **(A,B)** The expression level of TBULC in stable TBULC overexpression or mock vehicle control-transfected **(A)** A549 and **(B)** H226 cell clones. **(C,D)** The migration and invasion ability of TBULC-overexpressing **(C)** A549 and **(D)** H226 cell clones, as well as that of mock vehicle control-transfected cells, as detected by the Transwell assay. The numbers of migrating and invading cells were compared between the groups. **(E)** Representative images of lung tissues isolated from mice injected with 1 × 10^6^ A549 and H226 cell clones via the tail vein and hematoxylin and eosin-stained images (40×) of such tissues. Red arrows indicate the tumor nodules in the lungs. **(F)** The number of metastatic nodules in the lungs. **(G)** Relative expression levels of N-Cadherin, Slug, and Ras by Western blot in TBULC-overexpressing A549 and H226 cells. Tubulin was used as a loading control. The data are shown as the mean ± SD, ***p < 0.001.

To exclude the off-target effect of shRNAs and further clarify the effect of TBULC on cell invasion and migration, we established two NSCLC cell lines (A549 and H226 cells) stably overexpressing TBULC (Figures 3A,B). As expected, the invasion and migration abilities of A549 and H226 cells were significantly enhanced after upregulation of TBULC (Figures 3C,D). Collectively, these results demonstrated that TBULC has a positive regulatory effect on the invasion and migration of NSCLC cells. Furthermore, TBULC may be involved in NSCLC progression and metastasis by affecting NSCLC cell invasion and migration.

### TBULC Promotes NSCLC Metastasis *in vivo*

To further evaluate the role of TBULC in promoting metastasis in NSCLC, TBULC-overexpressing A549 and H226 cells were injected into the tail veins of non-obese diabetic nude mice. We calculated the number of pulmonary metastatic nodules in mice at 8 weeks after caudal intravenous injection. Mice injected with cells overexpressing TBULC showed higher rates of lung colonization and more metastatic tumor nodules in the lung than did mice injected with control cells (Figures 3E,F). H&E staining images of lung tissue samples isolated from mice are illustrated in Figure 3F. In addition, we found that TBULC significantly promoted the expression levels of N-Cadherin, Slug, and Ras, which belong to the TGFβ-related pathways and are widely involved in the metastasis of tumor cells (Figure 3G).

### TBULC Is Upregulated in NSCLC and Associated With Poor Survival

**Figure 4:**
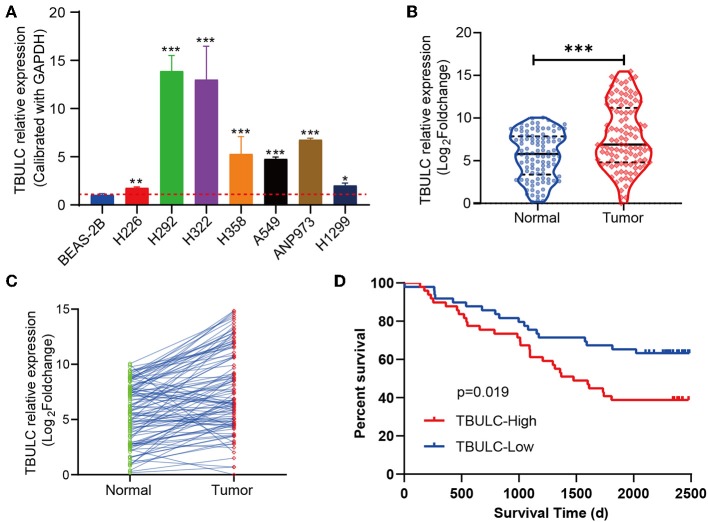
TBULC was upregulated in NSCLC tumor tissues and was associated with poor survival. **(A)** TBULC expression levels in the immortalized lung epithelial cell line BEAS-2B and seven NSCLC cell lines. GAPDH was used as the loading control. **(B,C)** TBULC expression (2–ΔCT) in 106 tumor tissues was compared with that in paired adjacent non-cancerous lung tissues. **(D)** Kaplan–Meier survival analysis of overall survival in 106 NSCLC patients (median cut-off value). The data are shown as mean ± SD, *p < 0.05, **p < 0.01, ***p < 0.001.

Previous functional experiments have shown that TBULC acts as an oncogene to promote NSCLC metastasis, while its expression pattern in NSCLC tumor tissues remains unknown. To further clarify its clinicopathological significance in NSCLC, RT-qPCR was used to detect the expression level of TBULC in 106 paired NSCLC and adjacent normal tissues, as well as in 7 NSCLC cell lines. Compared with that in the immortalized lung epithelial cell line BEAS-2B, the expression level of TBULC was significantly higher in all seven NSCLC cell lines (Figure 4A). Moreover, TBULC was significantly upregulated in NSCLC tumor tissues compared with paired normal lung tissues (*t* = 7.711, *P* < 0.001, Figures 4B,C). To determine whether TBULC affects patient prognosis, patients were divided into high and low expression groups according to the median expression of TBULC. There were no significant differences in age, sex, pathological types, or tumor stages between the two groups (Table 1, *p* > 0.05). The Kaplan–Meier survival curve and log-rank test indicated that lower expression of TBULC was significantly associated with better patient survival (X^2^ = 5.504, *p* = 0.019; Figure 4D). We used the TANRIC data base (https://ibl.mdanderson.org/tanric/_design/basic/query.html) to validate the impact of TBULC on prognosis. The results showed that high expression of TBULC in lung squamous cell carcinoma (*p* = 0.175) and lung adenocarcinoma (*p* = 0.082) suggests a poor prognosis, while the lack of statistical significance may be due to the limitations of RNA second-generation sequencing for relatively low-abundance lncRNA detection. In addition, the well-known NSCLC-associated lncRNAs MALAT1 and HOTAIR cannot be validated in the TANRIC database (Supplement Figure 1). Furthermore, a multivariate Cox regression analysis was applied to exclude the effects of clinical confounding factors, including age, TNM stage, lymph node metastasis, and degree of tumor differentiation, on patient prognosis. As shown in Table 2, TBULC was found to be an independent prognostic factor for NSCLC patients [*P* = 0.030, OR = 0.513 (0.281–0.936)].

**Table 1:** Correlations between clinicopathological features and the expression level of TBULC.

**Characteristics**	**TBULC relative expression**		**chi-square**	***p***
	**High expression (53)**	**Low expression (53)**		
**Gender**				
Male	37 (50.7)	36 (49.3)	0.044	0.834
Female	16 (48.5)	17 (51.5)		
**Age (y)**				
<60	29 (51.8)	27 (48.2)	0.151	0.697
≥60	24 (45.3)	26 (54.7)		
**Pathologic stage**				
Adenocarcinoma	27 (50.9)	26 (49.1)	0.038	0.846
Squamous	26 (49.1)	27 (50.9)		
**Pathologic grade**				
Poor	32 (54.2)	27 (45.8)	0.956	0.328
Medium and well	21 (44.7)	26 (55.3)		
**Lymph node metastasis**				
Positive	26 (50.0)	26 (50.0)	0.000	1.000
Negative	27 (50.0)	27 (50.0)		
**TNM stage**				
I, II	30 (50.8)	29 (49.2)	0.038	0.845
III, IV	23 (48.9)	24 (51.1)		
**Tumor size**				
<3 cm	17 (54.8)	14 (45.2)	0.410	0.522
≥3 cm	36 (48.0)	39 (52.0)		
**Smoking**				
<30	27 (48.2)	29 (51.8)	0.151	0.697
≥30	26 (52.0)	24 (48.0)		

**Table 2:** Univariate and multivariate analysis of overall survival in 106 NSCLC patients.

**Characteristics**	**Single-factor**	**Multi-factor**	**OR**	**95% CI**	
	***p***	***p***		**Lower**	**Upper**
Age (y)	0.049	0.096	1.631	0.917	2.901
TNM stage	0.008	0.198	1.906	0.714	5.089
Lymph node metastasis	0.04	0.876	1.081	0.406	2.883
Pathologic grade	0.012	0.210	0.657	0.34	1.267
TBULC	0.019	0.030	0.513	0.281	0.936

## Discussion

TGFβ family members and their downstream effector molecules have been widely demonstrated to be upregulated in various tumors, including NSCLC, and to play a vital role in tumor progression processes such as EMT, tumor immune escape, angiogenesis, chemoresistance, and metastasis (11, 13). However, because of the contradictory dual roles of the TGFβ pathway in tumor suppression and promotion, a large number of clinical trials targeting the key molecules of the TGFβ pathway and its downstream coding genes have failed (15). Therefore, the molecular mechanism by which TGFβ fulfills its role in either tumor promotion or suppression is key to the development of novel effective drugs targeting the TGFβ pathway. As a result of previous failures in the study of a large number of coding genes, in recent years, scholars have gradually turned their attention to lncRNAs, which were previously neglected. As a major component of non-coding RNAs, lncRNAs are a kind of RNA with a length of more than 200 bp that have no coding potential. A large number of studies have found that lncRNAs play an essential role in the occurrence and development of malignant tumors (14) and do not represent transcriptional noise. This study revealed that the novel lncRNA TBULC induced by the TGFβ classical pathway could promote the metastasis of NSCLC cells, which complemented the molecular mechanisms by which the TGFβ pathway promotes the local and distant metastasis of tumor cells. Moreover, TBULC is highly expressed in NSCLC tumor tissues and is associated with poor patient survival. Of note is that TBULC might be a novel prognostic biomarker in NSCLC patient prognosis.

In recent years, with the in-depth study of lncRNAs and the development of technology, many breakthroughs have been made in clinical treatment with lncRNAs (17). By utilizing subcutaneous injections of lncRNA MALAT1 translational oligonucleotides to treat breast cancer in mice, the Gayatri Arun team found that the suppression of MALAT1 by antisense oligonucleotides significantly inhibited the growth of mouse breast cancer and promoted tumor transition from solid nodules to cystic components; this approach could inhibit the invasion and migration ability of tumor cells *in vitro* in 3D-like organ cultures (16, 18). Owing to the high tissue and organ specificity of lncRNAs, drugs targeting lncRNAs have fewer side effects than drugs targeting coding genes. Our study is the first to demonstrate that the lncRNA TBULC is involved in the regulatory effect of TGFβ on NSCLC metastasis. Targeted drugs aimed at TBULC can bypass the TGFβ tumor suppressor pathway and may have fewer side effects. The lncRNA TBULC has the potential to become a novel drug target in NSCLC treatments.

There are several limitations to this study. Although the biological function of TBULC was verified by *in vivo* experiments, the specific molecular mechanisms of TBULC in promoting cell invasion and migration have not been well-studied. In addition, there was no statistical significance for TNM staging in the multivariate Cox regression analysis, which is probably due to the insufficient sample size and the loss of eight patients to follow-up. Additionally, the potential of targeted therapies such as TBULC oligodeoxynucleotide chains requires further exploration.

## Conclusion

In summary, the present study revealed that TGFβ-induced lncRNA TBULC was directly regulated by the classical TGF beta/Smad pathway and promoted the invasion and migration of NSCLC cells. This is an important supplement to the regulatory mechanisms of the TGF beta-pathway in tumor cell proliferation and metastasis. Moreover, in view of the high expression of TBULC in NSCLC tumor tissues and its independent prognostic significance, our study provided a novel biomarker for NSCLC prognosis as well as a potential drug target for NSCLC treatment.

## Data Availability Statement

All datasets generated and analyzed for this study are included in the article/Supplementary Material.

## Ethics Statement

This study was approved by the Ethics Committee of CICAMS, and all participants provided written informed consent.

## Author Contributions

SZ, ZL, CL, and XW performed the study design, experimental work, and manuscript drafting. RJ, SM, JHu, YL, and CZ performed the manuscript drafting and analysis. NS and JHe performed the experimental work.

### Conflict of Interest

The authors declare that the research was conducted in the absence of any commercial or financial relationships that could be construed as a potential conflict of interest.
