# Supplementary material for: The TGFβ-Induced Long Non-coding RNA TBULC Promotes the Invasion and Migration of Non-small Cell Lung Cancer Cells and Indicates Poor Prognosis
Source: Front Oncol. 2019 Dec 10;9:1340. doi: 10.3389/fonc.2019.01340 (PMC6914758; doi:10.3389/fonc.2019.01340)
Supplement: Supplementary file 1 [file Data_Sheet_1.docx]

Supplement

Figure 1 The survival curve of selected lncRNA in TANRIC database


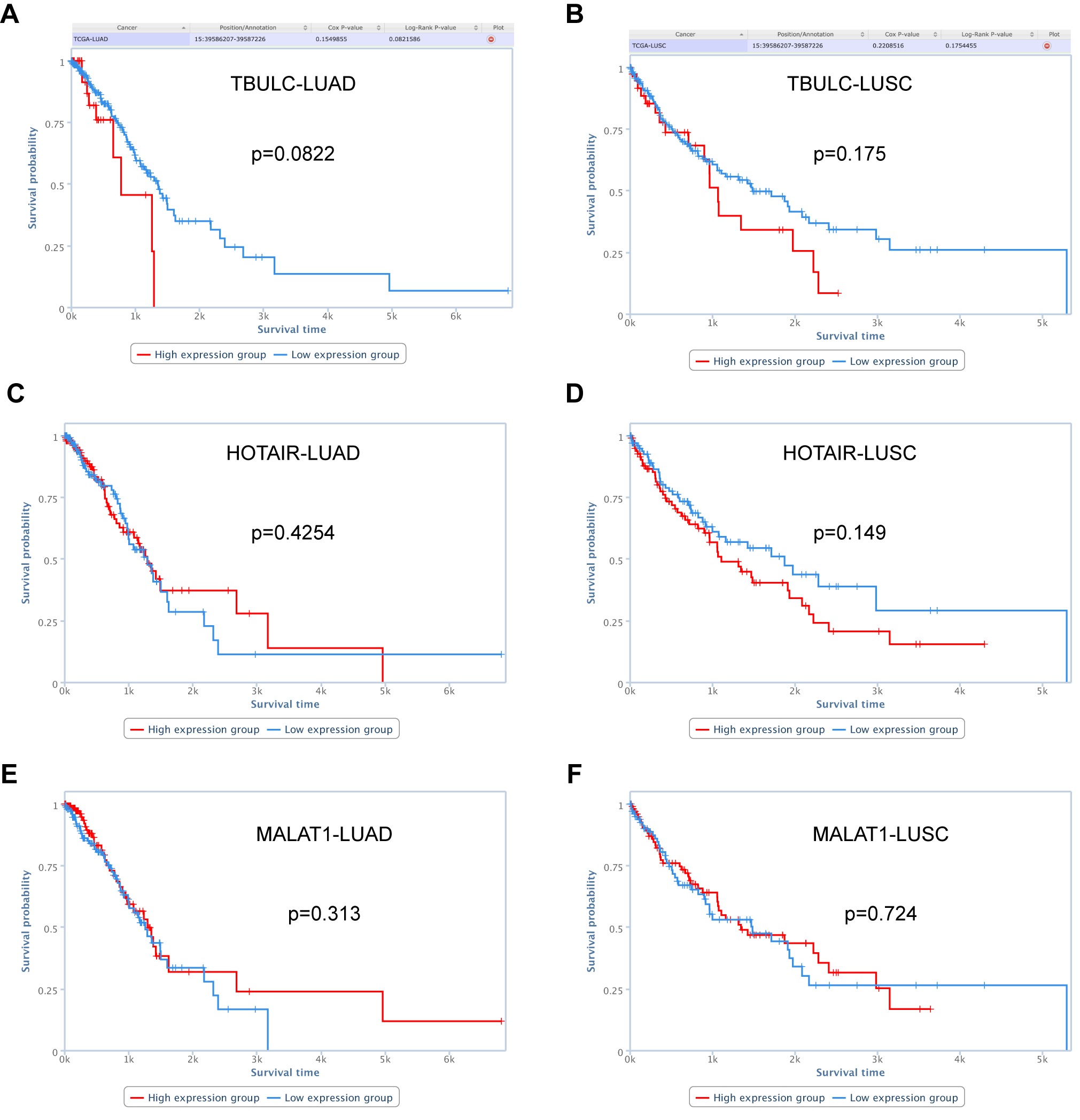


(LUAD, lung adenocarcinoma; LUSC, lung squamous cell carcinoma;)
